# Apoptosis Modulation as a Promising Target for Treatment of Systemic Sclerosis

**DOI:** 10.1155/2011/495792

**Published:** 2011-09-06

**Authors:** Stéphane Chabaud, Véronique J. Moulin

**Affiliations:** ^1^Centre LOEX de l'Université Laval, Génie Tissulaire et Régénération, LOEX-Centre de Recherche FRSQ du Centre Hospitalier Affilié Universitaire de Québec, Quebec City, QC, Canada G1J 1Z4; ^2^Département de Chirurgie, Faculté de Médecine, Université Laval, Quebec City, QC, Canada G1V 0A6

## Abstract

Diffuse systemic sclerosis (SSc) is a fatal autoimmune disease characterized by an excessive ECM deposition inducing a loss of function of skin and internal organs. Apoptosis is a key mechanism involved in all the stages of the disease: vascular damage, immune dysfunction, and fibrosis. The purpose of this paper is to gather new findings in apoptosis related to SSc, to highlight relations between apoptosis and fibrosis, and to identify new therapeutic targets.

## 1. Introduction

Systemic sclerosis (scleroderma, SSc) is a heterogeneous disease which develops into three forms: limited, intermediate, and diffuse [1]. The limited form only affects skin of the limbs. In addition to cutaneous disorders, the diffuse one also affects internal organs such as lungs, heart, and kidneys. After a debilitating phase, the most severe form leads to death. This disease is characterized by a strong autoimmune reaction, although it is not clear whether this is a consequence of the disease or a causal factor. Nevertheless, autoantibodies, principally raised against nuclear epitopes, are used like prognostic markers. 

In the United States, the disease strikes more African American people and females than Caucasians and males [2]. Besides, the disease appears more prematurely in the African American group and hits them more severely than the Caucasians [3]. The mortality rate for SSc in the group of women has increased by seventy percent over the last twenty years without convincing explanations. The cost of the medical care associated with this disease was estimated at more than $20,000 per patient per year in Canada [4]. The disease is particularly devastating because it strikes people during the most productive period of their professional life.

 The causes of SSc are not clearly identified. Genetic factors could not be excluded [5, 6], but environmental influence seems to be more important. Notably, chronic exposition to chemicals, such as organic solvents [7] and silicone [8], viral infection by cytomegalovirus (CMV), a member of herpesviruses family [9, 10], and microchimerism [11], could all play a role in the disease.

It is thought that SSc begins with vasculopathies through massive endothelial cells death that would lead to obliteration of small arteries and arterioles. It is however unclear if autoantibodies are produced before vascular damages and/or in response to it. Subsequently, cell-to-cell communications are substantially altered, notably by cytokine and growth factor secretion dysregulation. The consequence of these biological changes is an excessive extracellular matrix (ECM) deposition, fibrosis, in tissues followed by their loss of function [12]. SSc varies during the progression of the disease, showing noteworthy changes in fibroblasts phenotype [13]. At the early stage of the disease, cells respond to TGF*β*, but become totally insensitive at the late stage for the most affected patients, explaining why treatments targeting this cytokine remain mostly ineffective.

Over the last decades, various models had been used to study SSc [14]. Some animals develop a disease similar to this pathology such as UCD-200 chicken, Tsk-1 and -2 mice. Normal mice could also show some symptoms of the disease after a treatment with Scl-GvHD, Bleomycin, or growth factor injection. Nevertheless, several observations made on these animal models are quite different than those ascertained in humans. Primary cultures of cells isolated from patients are also widely used, and, more recently, a new tissue-engineered reconstructed skin model was shown as a powerful tool to assess the mechanisms involved in the progression of human SSc [13].

## 2. A Brief Overview of the Mechanisms of Apoptosis

From the development of the embryo up to its death, apoptosis plays a crucial role in the induction and the maintenance of several physiologic functions, at several steps of both normal and atypic development steps during life. By eliminating cells during the development of the embryo, it assists at leading the modeling patterns of the body. It also contributes to eliminating the nonfunctional neurons and allows the selection of the adequate synaptic connections. Later, it allows the elimination of unsuitable lymphocytes (AICD, activation-induced cell death) [15], preservation of the homeostasis [16, 17], contributing to the maintenance of the functional status of the immune system by eliminating deviant or infected cells. However, an unbalanced ratio between the various factors involved in apoptotic pathways can lead to excessive cell proliferation and potentially to cancer or, on the contrary, to neurodegenerative diseases (Alzheimer, Parkinson), autoimmune diseases (rheumatoid arthritis), or immunosuppressive diseases (AIDS).

Apoptosis, or programmed cell death, should not be confused with necrosis. The apoptotic process occurs according to specific and sequential steps that have been documented. In contrast, necrosis results from passive mechanisms that lead generally to an inflammatory process while apoptosis does not [18]. Apoptotic cells are truly destroyed from the inside and quickly removed from the tissues, a situation that seriously delayed the discovery of this physiologic process [19]. Apoptosis is regulated through a sequence of events that have been described. First, the cellular morphology changes with the formation of structures in bubbles, rounding of cells probably related to a disorganization of actin filaments, a reduction of the cytoplasmic volume, a weakening of mitochondrial membrane, facilitating the liberation of various factors. The chromatin condenses, and the DNA is cleaved at the level of nucleosomes, before the fragmentation of the cell nucleus. Finally, the cells are fragmented into apoptotic bodies that are mainly cleared by macrophages.

**Figure 1:**
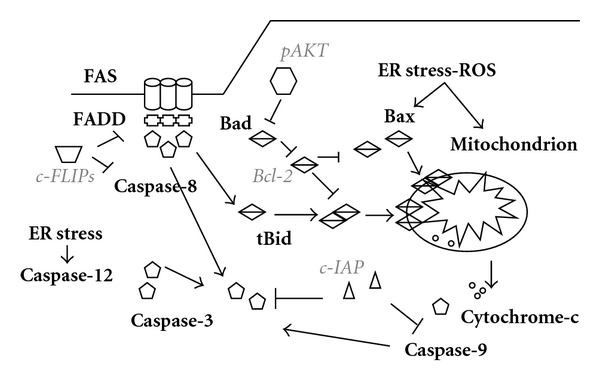
Major apoptotic pathways. Schematic representation of intrinsic, mitochondrial, and extrinsic, death receptor, pathways. Proapoptotic molecules are **in bold** and antiapoptotic are *in italic*.

Several mechanisms are described as playing a role in the induction of apoptosis [20]. Following a proapoptotic stimulus, a cascade of specific proteases is activated in the cell. These proteases are called caspases, cysteinyl aspartic acid proteases [21]. Caspases are produced as proenzymes which must be cleaved to become active. Protease specifity is defined for each caspase by an aspartic acid containing consensual sequence in target proteins. Caspases involved in apoptosis could be classified in 2 groups: the initiator or apical caspases, like caspase-8 and caspase-9, whose role is to activate the other caspases, and the effector or downstream caspases, like caspase-3, which generally cleave vital targets. Apoptotic pathways differ according to the nature of the inductor and the cell type, making their study very complex, especially in vivo. Nevertheless, two main pathways can be distinguished: the mitochondrial, or intrinsic, pathway and the cell death receptors, or extrinsic, pathway (Figure 1). There are numerous connections between these two pathways, then it could be difficult to separate them. Other pathways have also been described, but would be of lesser importance.

The mitochondrial pathway seems to be the most common apoptotic mechanism, and it is well documented [22]. Under the influence of several stimuli (ultraviolet irradiations, chemical damage, etc.), proteins of the Bcl family act independently or in complexes on the mitochondrial membrane [23, 24]. Bcl family proteins could be separated in proapoptotic proteins (Bax, Bak, Bok, Bcl-Xs), which destabilize the mitochondrial membrane, antiapoptotic proteins (Bcl-2, Bcl-XL, Bcl-W, Mcl-1, Bcl-2A1), that protect this membrane and proapoptotic BH3-only proteins (Bad, Bid, Bik, Bim, Puma, Noxa), which inhibit the antiapoptotic function of the Bcl-2-like proteins [25]. Under Bcl proapoptotic protein influence, mitochondrial pores open and lead to depolarization of the membrane releasing proapoptotic factors such as cytochrome C. The latter associates with the adaptor Apaf-1 (apoptosis-associated factor-1) and procaspase-9. In presence of dATP, this complex, named apoptosome, leads cleavage and activation of procaspase-9 which responds by cleaving procaspase-3 to activate it. Caspase-3 degrades its substrates such as ICAD/DFF45, an inhibitor of the endonuclease CAD/DFF40 (caspase associated DNAse), responsible for the fragmentation of the DNA. Various inhibitors exist for this pathway such as some antiapoptotic proteins of the Bcl family [23], preventing the depolarization of the mitochondrial membrane. Furthermore, when phosphorylated by AKT/PKB, Bad, a proapoptotic member of this family could be sequestrated by the protein 14.3.3 [26]. This action prevents Bad to bind to Bcl-xL and inactivate its protective function. IAP (inhibitor of apoptosis protein) could also block mitochondrial apoptosis by inhibiting the cleavage of procaspases 3, 7, and 9 [27, 28]. Other pathways that are dependent from mitochondria but independent from caspases can also be activated, notably by the factor AIF (apoptosis inducing factor) released from mitochondria [29]. 

The second best known apoptotic pathway, the cell death receptor (DR) pathway, has also been extensively studied [30–32]. Until now, about fifteen DRs from the TNF (tumor necrosis factor) receptor family were identified. Some are well known, especially TNFR-1/p55 and Fas/Apo-1/CD95. They are characterized by an extracellular domain consisting of cysteyl-rich regions (from 1 to 6 repeats), a transmembrane domain, and an intracellular domain carrying a death domain region (DD). Inserted in the plasma membrane of the cells, monomeric or trimeric forms of receptors are clustered into microdomains called lipid rafts [33–35]. The DR ligands are mostly transmembrane proteins that must be matured by proteolytic cleavage [36]. Once released, they could bind their receptors although it has been shown that Fas ligand (FasL) and the membrane form of the TNF (mTNF which is especially bound to TNFR-2 [37]) can also act in a transmembrane form. Activation of TNFR and Fas [38] induces a wide range of functions such as cellular proliferation, cell survival and apoptotic cell death, cell differentiation, inflammation, and defense against microorganisms (viral, bacterial, fungal, and parasitic infections) [39]. Output of ligand binding is determined by cellular environment and adapter molecules availability. 

According to the cells, mechanisms to induce apoptosis via DR pathway are different. In type-I cells, proapoptotic cascade is fast and unaffected by a Bcl-2 overexpression, while, in type-II cells, apoptotic cascade is slower, demonstrates an activation of procaspase-9, and is sensitive to Bcl-2 antiapoptotic activity [40]. In type-I cells, such as dermal fibroblasts, Fas activation by FasL leads to the recruitment of FADD (Fas-Associated DD), an adapter protein [41]. FADD shares homologous domains with its partners: DD with receptor and Death Effector Domain (DED) with apical caspases. FADD allows the recruitment and self-activation of procaspase-8, which can consequently cleave and activate procaspase-3. This pathway is tightly modulated by c-FLIP (cellular-flice inhibitory protein) [42, 43], a caspase homolog without any proteolytic activity. cFLIP can bind FADD or procaspase-8 and prevents its activation. Once more, IAP could inhibit caspase-3 activity. 

Mechanisms associated to apoptosis of type-II cells seem, at least partially, common with the mitochondrial pathway. In these cells, caspase-8 activity is weak, and one of its targets is Bid, of which the truncated form, t-Bid, acts on mitochondria [44].

Mitochondrial and DR pathways could be regulated by the activation of the MAPK (Mitogen-Activated Protein Kinase) cascade [45]: p38 MAPK, ERK (Extracellular signal-Regulated Kinase) and JNK (Jun N-terminal Kinase) or by those of NF*κ*B (Nuclear Factor-*κ*B) [46]. MAPK activation could lead to survival or apoptosis, depending on the strength and duration of the signal. The antiapoptotic function of these kinases mostly results from transcription factor activation, leading to antiapoptotic proteins synthesis, notably from the Bcl family. NF*κ*B exists on an inactive form linked to inhibitor I*κ*B. Phosphorylation and subsequent degradation of this protein release a phosphorylated form of NF*κ*B which translocates to the nucleus and activates not only the expression of c-FLIP [47], but also the expression of c-IAP1 and 2, XIAP, and other various antiapoptotic proteins.

Although less studied, other apoptotic pathways are documented. Cytolytic granules are specialized secretory lysosomes mainly composed of perforin and granzymes. Granules are secreted by killer cells, such as natural killer (NK) or cytotoxic T lymphocytes (CTLs) to control viral infection, intracellular pathogen dissemination, and tumorigenesis. After granule is released in immunological synapses, perforin opens cell membrane allowing entry of granzyme into the cell using a still unclear mechanism. Granzyme B cleaves and activates not only procaspase-3 but also Bid in its t-Bid form and ICAD to release DNAse CAD. Granzymes A and C do not activate procaspases but act on mitochondrial membrane to induce apoptosis. Granzyme A could also target SET complex in endoplasmic reticulum which in turn induces DNA damage [48, 49]. Endoplasmic reticulum (ER) cell responses are important to appropriately answer to unfolded proteins but, when unresolved, they could induce apoptosis [50, 51]. ER stress induces apoptosis through mitochondria or activation of procaspase-12 and subsequently procaspase-3 [52]. 

Our aim was not to give an exhaustive description of apoptosis, and we voluntary restrain it to major pathways. Very interesting reviews could be read on other forms of cell death, especially necrosis, oncosis [53], and necroptosis, a well-organized death [54]. 

## 3. Fibrosis and Apoptosis

Links between apoptosis and fibrosis emerged with force during the late 1990s. The level of the soluble form of Fas, sFas (a receptor antagonist for the proapoptotic protein Fas [55]), in sera from affected patients is higher than the one of normal volunteers in idiopathic pulmonary fibrosis [56] and silicosis [57]. This increased sFas serum level could play a role in preserving a subset of dysfunctional lymphocytes. Activated but unwanted lymphocytes which are normally eliminated by Fas activation could remain and induce fibrosis. They could also increase the fibroblastic cellularity and consequently the collagen deposition. The Fas/FasL system in T cells is also altered after silica exposure and leads to silicosis [58]. Whereas pulmonary fibrosis involves an increased resistance to apoptosis [59, 60], Fas is downregulated in fibrotic fibroblast membrane [56] and the proapoptotic Bid was shown to be required to induce fibrosis [61]. As in silicosis, the Fas/FasL system was thus thought to play a key role in several other pulmonary fibrotic pathologies [62, 63].

Similar results were observed in SSc. For instance, an increased level of sFas in SSc serum [64, 65] and a higher resistance of pathological fibroblasts to Fas apoptosis [66–69] have been reported. Some studies with bleomycin-treated mice indicated that the Fas/FasL pathway is critical for the development of SSc pathology. Animals devoid of Fas or FasL genes [70] or treated with anti-FasL antibodies [71] show a decrease of apoptosis and a concomitant decrease of collagen accumulation. SSc affects the immune system of the patients since changes in Th1/Th2 response were detected resulting in an inadequate profibrotic activation [72–77]. Several authors attribute the antifibrotic effect of the Fas pathway deletion to a decreased selection of altered profibrotic lymphocytes subset. The selection of these abnormal lymphocytes would mainly result from a deficient apoptotic process [78, 79]. It could also be noted that Th cells have different sensitivity from Fas-induced apoptosis and that difference could be explained by cFLIP expression level. Th2 and Th17 are naturally more resistant to Fas-induced cell death than Th1 [80]. Fas activation could then lead to selection of a Th2/Th17 response to the detriment of Th1. This is the immunologic pattern generally expected in SSc. Overexpression of FLIP decreases sensitivity of all these cells [81] and could reverse autoimmune disease in animal model [82]. sFas levels could then be an attempt to patient to stop the Th1 to Th2/Th17 fibrotic switch. 

SSc also affects the vascular network and a lot of evidences pointed out apoptosis as an effector. Sera from patients have been shown to induce apoptosis according to several mechanisms. Antiendothelial cells antibodies (AECAs), a heterogeneous group of antibodies directed against proteins and molecules specifically present on surface and inside of endothelial cells, induce apoptosis by stimulating Fas [83] or activating procaspase-3 [84]. Apoptosis could also be amplified when a patient is infected by CMV, one of the putative cause of SSc. This amplification was done via viral UL94 protein [85]. In SSc patients, sera could also induce endothelial apoptosis via secretion of IL-6 by monocytes (or fibroblasts) and E selectin expression [86]. In healthy people, a break in the vascular network results in a quick repair and the reconstitution of small vessels via angiogenesis. In SSc patients, this repair is blocked by the death of both, endothelial progenitor cells (EPCs) and circulating angiogenic cells (CACs). Death of EPC results from factors that are present in SSc patient sera such as AECA [87]. In EPC exposed to SSc patient sera, pAKT is reduced and could not inhibit the phosphorylation of FOXO3a and Bim, a proapoptotic Bcl-2 family member, is increased [88]. Furthermore, authors have demonstrated that CAC are killed by microparticules (MPs) released by apoptotic endothelial cells. These MP membranes are rich in arachidonic acid, which induces mitochondrial death of CAC [89, 90]. Tweak, known for its capacity to preserve and develop vascular network, is decreased in SSc patient sera with pulmonary affection [91], but not in sera of SSc patients with unaffected lungs [92]. This impairment of vascular endothelial repair results in hypoxia, which, in turn, could induce apoptosis in immune system cells and fibroblasts. Together with profibrotic IL4 cytokine, widely expressed in SSc, hypoxia also results in an increase in Lysyl-hydroxylase-2 and an alteration of the type-I collagen crosslinking [93]. Hypoxia also helps to stimulate ECM deposition, and, in a vicious loop, such tissue fibrosis induces more hypoxia [94]. Apoptosis of endothelial cells has other effects, notably on fibroblasts, discussed in the next paragraphs. 

Some evidence indicates apoptotic epithelial cells could play a role in fibrosis, especially in lung of bleomycin-treated mice [95, 96] as well as in a new SSc mouse model [97] and human Idiopathic Pulmonary Fibrosis [98]. Epithelial cell death is clearly mediated through Fas/FasL pathway. Activated T cell and fibrotic fibroblasts could express FasL and induce apoptosis in lung epithelium [99]. During repetitive cycles of epithelial injury, epithelial cells release cytokines and growth factors, which promote fibroblast activation such as in wound healing process. These chronic phenomena lead to fibrosis. 

Recent data indicate that SSc patient epidermis is abnormal and play a role due to its interaction with dermis [100, 101] like it was shown previously in skin wound healing [102]. SSc keratinocytes promote release of TGF*β*, a fibrosing agent, from fibroblasts and secrete themselves CTGF, which stabilize the fibrotic phenotype of fibroblasts. Change in epidermis-dermis interaction in SSc is thought to result from chronic epidermis injury. The exact cause of this injury remains unclear but a limited apoptosis could not be excluded at this level, especially due to results obtained in lung fibrosis.

Fibroblasts, the major ECM secreting cells, also play a role in the evolution of SSc. Many studies focus on the regulation of fibroblast apoptosis in relation with fibrosis. It is thought that profibrotic cells are more resistant to apoptosis than others, maintaining their presence in situ despite immune system endeavour to remove them. This has been observed in various types of fibrosis such as hypertrophic scars [103, 104], pulmonary fibrosis [59, 60], and SSc [66–69]. Proteins from the mitochondrial pathway seem to be involved in the apoptotic resistance as well as death receptors proteins and several elements connecting both pathways. Some studies highlight proteins of mitochondrial pathway. Bax expression is decreased in SSc dermal fibroblasts [69] but Bcl-2 could be increased [105]. pAKT is increased in SSc fibroblasts [105, 106] and then could inhibit Bad proapoptotic function. The Fas pathway is also repressed in SSc with c-FLIPs and through c-IAP overexpression [67]. In other studies, protective potential of SSc fibroblast is explained by modulation of transcription of antiapoptotic proteins through kinase cascade. MAPK pathway, including ERK, is also activated by pFAK [107, 108]: the result of such activation could lead to the expression of antiapoptotic proteins. MIF [105], an ERK activator inducing Bcl-2 expression, and AKT, or PKC*ε* [66], responsible for MAPK activation, are both reported to be involved in the modulation of apoptosis in SSc fibroblasts. Involvement of ROS in SSc is known for a long time [109]; they result mainly from vascular damage and inflammatory process. Besides protein and lipid oxidation, they induce DNA damage in fibroblasts [110] but seem to play little or no role in SSc-related vasculopathy. It is however well known that DNA damage could, in turn, induce apoptosis.

## 4. Selection of Profibrotic Fibroblast Populations: Role of Apoptosis

Several groups have postulated that lesional SSc fibroblasts could have been selected through unknown mechanisms from a subpopulation already present in situ, prior to the emergence of the first lesions. 

Fibroblasts are heterogeneous in terms of their collagen secretion pattern [111] that they retain for several passages in vitro [112]. In SSc lesions, there is an increase in fibroblasts producing high levels of collagen and this phenotype is also retained in vitro [111–113]. A clonal selection of high-collagen-producing fibroblasts had been proposed as a mechanism for scleroderma-associated fibrosis onset [114]. It is postulated that the increase of the high collagen producing cells in SSc tissues could result either from a higher proliferative capacity or from a higher resistance to apoptosis of these cells. Some authors have shown that an exposure of fibroblasts to SSc sera increases the proportion of fibrotic fibroblasts [112], but others do not [115]. Lesional SSc fibroblasts do not grow at higher proliferation rates than normal ones in monolayers [67] or in three-dimensional tissue engineered cultures [13]. Recent evidences point out apoptosis resistance as the main mechanism from which profibrotic/apoptosis-resistant cell subpopulations emerge at the detriment of healthy cells leading to the development of fibrotic lesions. Fibroblasts from nonlesional area of late stage SSc patients exposed to FasL show an increased resistance to Fas-induced apoptosis and a decrease of their MMP secretion, which could result in higher ECM deposition. A similar mechanism was described for metastatic cancer cells [116]. 

## 5. Are Apoptosis Resistance and Profibrotic Potential of Fibroblasts Related to or Resulting from Independent but Concomitant Mechanisms?

During SSc development, fibroblasts are surrounded by numerous proapoptotic and profibrotic stimuli [76, 115, 117]. It could be interesting to relate both events. After vascular damage, thrombin is released in order to form a fibrin clot, but it also activates PAR-1, inducing the modulation of the fibroblast phenotype from quiescent to fibrotic. At the same time, thrombin promotes fibroblast apoptosis resistance through the effect of p21Cip1/WAF1. This protein induces PKC*ε* activation that inhibits Fas/FasL signaling and reduces or slows down apoptosis [66]. Endothelial cell death leads to the local diffusion of several mediators including not only CTGF [118], a protein involved in the stimulation of fibrosis [119], but also some antiapoptotic factors that may promote the maintenance of lesional SSc fibroblasts on site [120]. Various types of cells died through apoptosis during SSc, probably due to patient sera composition, FasL exposition, or hypoxia. Apoptotic bodies released from such dead cells may contribute to the survival of fibroblasts that exhibit a profibrotic phenotype in response to macrophages TGF*β* secretion [121]. It is interesting to note that Thrombospondin-1 (TSP-1) is increased in SSc and activates the release of TGF*β* from latent complex [122]. TSP-1, released from apoptotic fibroblasts, is also responsible of the activation of apoptotic body phagocytosis by macrophages [123]. Some microRNAs recently discovered also seem to play a role in the potential interactions between apoptosis and fibrosis. Mir-29b decreases the expression of Mcl-1, a Bcl-2 family member [124], sensitizing cells to apoptosis. Mir-29a has been demonstrated to repress collagen synthesis, and it is significantly poorly detected in SSc fibroblasts [125]. Finally, Mir-29a/b transcription is modulated by NF*κ*B [126], a protein with dual roles in apoptosis.

**Figure 2:**
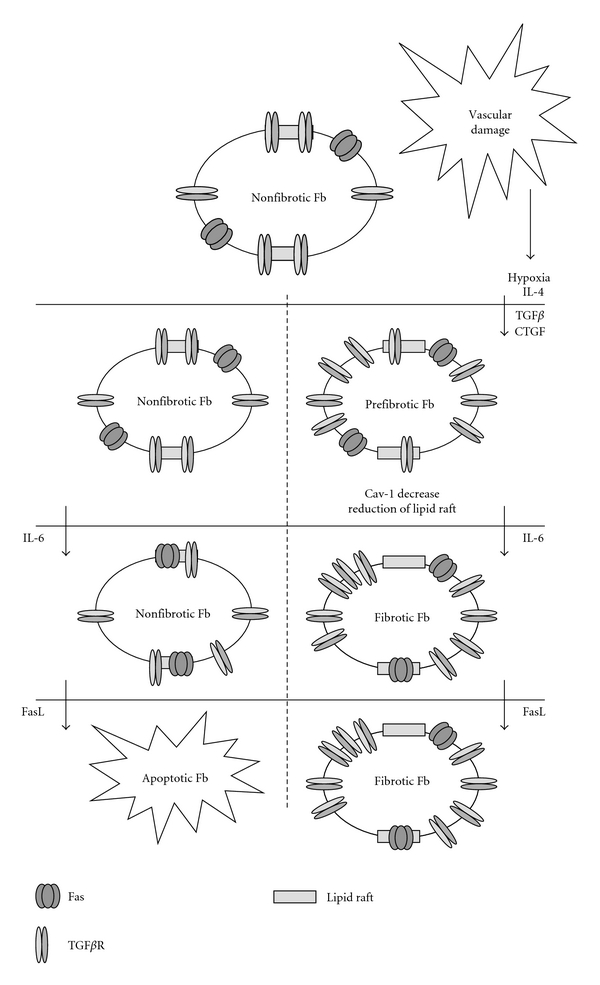
Hypothetic mechanism of FasL/IL6 selection of prefibrotic fibroblast in scleroderma lesions. After vascular damage, various factors are directly secreted by apoptotic endothelial cells or are secreted by macrophages, T cells, and fibroblasts in response to endothelial cell deaths and subsequent hypoxia. More proximal cells become prefibrotic by increasing their response to TGF*β*. IL-6 secretion induces a more fibrotic phenotype for cells and increases apoptosis-resistant feature of profibrotic ones. FasL secreted by infiltrating cells kills nonfibrotic fibroblast with low or none effect on fibrotic cells. ECM is then deposited in excess and organ fails.

Nevertheless, the protein that seems to be the most relevant to link apoptosis and fibrosis is IL-6. IL-6 is strongly overexpressed in SSc [76, 86, 117, 127–131]. This cytokine is known to induce a relocalization of receptors outside lipid rafts (Figure 2). This change of compartmentalization increases TGF*β* signaling and collagen synthesis [132]. Interestingly, in response to IL-6, fibrotic cells become more resistant, and normal fibroblasts become more sensitive to apoptosis [133]. It is not known if IL-6 can change Fas localization outside lipid rafts in fibrotic cells and inside the rafts in normal cells. However, several authors have demonstrated that translocation of Fas in lipid rafts increases cellular response to apoptosis [134–139]. 

## 6. New Therapeutic Targets

No treatment is currently available to help SSc patients who deal with a major loss of function and a poor quality of life. A deeper knowledge of the mechanisms underlying SSc is thus required to identify new therapeutic targets to establish a therapeutic strategy to control or cure SSc [140]. As the primary causes of the disease are unknown, it is necessary to target the secondary causes. Apoptosis is the heart of the SSc. In every stage, establishment and maintenance of the disease, this phenomenon plays a crucial role. So, it should be possible to modulate apoptosis to block the development of the disease and, perhaps, to go back towards a healthy status especially for early stage of the disease. 

### 6.1. Targeting Immune System Cells Apoptosis

In SSc, Th balance is in favor of Th2–Th17 cells allowing secretion of profibrotic cytokines as TGF*β* and IL6 rather than Th1 cells, TNF*α* secreting cells, which are less harmful. Sensitivity of Th cells to Fas-induced apoptosis favors clonal selection of Th2–Th17 cells, which express more cFLIP than Th1 cells. Overexpression of cFLIP in a transgenic mouse model leads to restoration of Th balance [82]. Nevertheless, cFLIP accumulation should be restrained to lymphocytes because cFLIP presence also promotes fibroblast resistance to Fas-induced apoptosis and selection of fibrotic dermis cells [67]. Gene therapy could achieve this goal but no safe and efficient gene transfer protocol is available for now. 

It had been observed that SSc patient peripheral blood mononuclear and T cells secrete more sFas, the soluble form of Fas, to trap FasL, and thus to reduce apoptosis in tissue. It is believed that this strong sFas secretion could result in a selection of an unwanted subset of profibrotic-activated lymphocytes, but in the light of the results mentioned below, it also could be an unsuccessful attempt to restore Th balance. In this case, treatment with recombinant sFas could favor Th1 cells. Injection of blocking but not activating anti-Fas antibodies or anti-FasL could also be envisaged [71]. In the same vein, downregulation of Fas exposition at the surface of the cells [141] or a decrease of the Fas concentration in lipid rafts could also be tested [138, 142]. As it has been shown in transgenic mouse model where Fas or FasL genes were deleted, Fas/FasL pathway abolition could thus result in an abrogation of fibrosis development [70]. 

Finally, several studies link breast cancer and scleroderma [143]. Tamoxifen was known to restore Th1/Th2 balance [144] and could be a candidate to potentially block the disease evolution. 

### 6.2. Targeting Endothelial Cells Apoptosis

Vasculopathy is an early event in the development of SSc, and a lot of patients received their SSc diagnostic when they consult for Raynaud-like symptoms. Then, endothelial cell apoptosis managing could be a valuable strategy. At this level, protecting endothelial cells or their precursors is a need. As previously described, SSc pathologic process targets not only endothelial cells from microvessels but also the mechanism to repair the vascular damage. 

Endothelial cell apoptosis is induced by pathologic serum exposition especially AECA. Two strategies could be developed: to determine what are the antigens recognized by these antibodies and to inject recombinant peptides to block their action or to design antibodies raised against AECA to inactivate them. The initial vasculopathy blocking should then stop the disease at a very early stage. 

In order to allow an efficient repair of microvessel, restoration of microcirculation, and reversion of fibrosis, it is also required to prevent EPC and CAC death. Arachidonic acid containing MP could be counteracted by Oltipraz and 1,2-dithiole-3-thione congeners [145]. VEGF and/or PDGF treatment could also been envisaged to maintain or restore microvasculature. This therapy should however be coupled with another one that can block endothelial cell apoptosis. 

### 6.3. Targeting Epithelial Cells Apoptosis

Apoptosis modulation in the lung epithelial compartment could be achieved by reducing exposition of FasL to the surface membrane of activated fibroblasts or to decrease Fas at the surface of the lung epithelium. Epithelia are very accessible tissue to therapy due to their localization in direct contact with outside. Gene therapy assay to reduce Fas expression should thus be easily driven.

### 6.4. Targeting Fibroblasts Apoptosis

Because fibroblasts secrete ECM responsible of fibrosis, these cells should be targeted as soon as first fibrotic symptoms are obvious. The challenge of this step is the very heterogeneous molecular results at the origin of fibroblast resistance to apoptosis. The diversity of the mechanisms, and then of the antiapoptotic proteins involved, could result from the various disease aetiologies, the ethnic patient origins, or the model used. Nevertheless, a precise definition of the apoptotic mechanism involved for each patient is clearly needed.

Hyaluronan could be a valuable molecule to treat this pathology. Hyaluronan decreases collagen synthesis by reverting localization of TGF*β* receptors in nonsignaling membrane domain [146]. It also induces translocation of Fas into lipid rafts and then sensitizes cells to apoptosis [136]. Similar effects on Fas recruitment in lipid rafts are obtained with edelfosine, an inhibitor of PI3K [33, 134, 135, 137]. 

In the same way, restoring expression of Caveolin-1 could be of a great help. Cav-1 expression is reduced in SSc [147], as well as in some breast cancers [148], and this decrease favors localization of TGF*β* receptors outside lipid rafts and towards signalling domains. Besides, Fas is effective to induce apoptosis when presents in lipid rafts. So Cav-1 underexpression could explain, at least partially, the resistance of fibrotic fibroblasts to Fas-induced apoptosis. Restoration of Cav-1 expression level could thus help to reverse fibrosis.

Curcumin could also provide a promising strategy for the treatment of SSc. Curcumin has been shown to induce apoptosis in fibrotic cells only [149]. This molecule is widely used in traditional south-east Asian cooking and for cancer treatment [150–154]. It is a safe drug but its optimal effect is obtained when administered in combination with other drugs. Another natural product, Resveratrol, has proapoptotic properties which could be used to sensitize fibrotic cells to apoptosis [155, 156]. This molecule inhibits Mcl-1 and Bcl-XL which are both known to play a role in apoptotic resistance of fibroblasts in SSc. Resveratrol also counteracts AKT and MAPK pathways. Regulation of oxidative stress has been extensively previously described and may help to define antioxidant therapy to restore normal function of organs including skin [157]. 

In order to increase Fas sensitivity of fibrotic cells, thalidomide could be used. Thalidomide has been shown to increase Fas number [158]. Presence of infiltrating T cells producing FasL in SSc tissues could thus result in the elimination of fibrotic cells. Thalidomide is currently used for the treatment of autoimmune diseases [159] inducing apoptosis through an unknown mitochondrial pathway [160]. Restored levels of mir-29a/b could also be of a great help to control scleroderma but further studies based on microRNA seem to be necessary. Sexual hormones such as oestradiol and testosterone could also modulate the expression and the sensitivity of cells to the FasL/Fas pathway [161]. As the majority of SSc patients are women who have reached the end of their active reproductive period, the potential adverse effects of hormonal treatment could be minimized, with a rigorous clinical monitoring. Control of cFLIP and cIAP expression using siRNA or antisense oligonucleotide could also be of interest [67]. Anticancer therapy targets these proteins [162]. In the case of fibroblasts, cFLIP expression level need to be decreased in fibrotic cells or increased in their nonlesional counterparts. cIAP is a more complex protein to modulate due to its redundant isoforms but could not be eluded due to its numerous biological functions and its potential role in SSc.

## 7. Concluding Remarks

Apoptosis plays a key role in emergence and maintenance of SSc. Apoptosis is involved in immune system response by changing the subset of infiltrating cells, in vascular damage and its consequences, and finally in selecting fibroblasts with profibrotic phenotype, responsible of loss of function of organs and fatal outcome. Depending on the patient, molecular mechanisms could be different and involve different proteins. Nevertheless FasL/Fas seems to play a key role. A deeper knowledge of how apoptosis modulates fibrosis could allow the development of new therapies adapted to the apoptotic profile of patients and, thus, to cure the disease with the least adverse side effects.

## References

[1] Lonzetti LS, Joyal F, Raynauld JP (2001). Updating the American College of Rheumatology preliminary classification criteria for systemic sclerosis: addition of severe nailfold capillaroscopy abnormalities markedly increases the sensitivity for limited scleroderma. *Arthritis and Rheumatism*.

[2] Krishnan E, Furst DE (2005). Systemic sclerosis mortality in the United States: 1979–1998. *European Journal of Epidemiology*.

[3] Laing TJ, Gillespie BW, Toth MB (1997). Racial differences in scleroderma among women in Michigan. *Arthritis and Rheumatism*.

[4] Bernatsky S, Hudson M, Panopalis P (2009). The cost of systemic sclerosis. *Arthritis Care and Research*.

[5] Arnett FC, Howard RF, Tan F (1996). Increased prevalence of systemic sclerosis in a native American tribe in Oklahoma: association with an Amerindian HLA haplotype. *Arthritis and Rheumatism*.

[6] Liakouli V, Manetti M, Pacini A (2009). The 2670G>A polymorphism in the FAS gene promoter region influences the susceptibility to systemic sclerosis. *Annals of the Rheumatic Diseases*.

[7] Garabrant DH, Lacey JV, Laing TJ (2003). Scleroderma and solvent exposure among women. *American Journal of Epidemiology*.

[8] Brinton LA, Buckley LM, Dvorkina O (2004). Risk of connective tissue disorders among breast implant patients. *American Journal of Epidemiology*.

[9] Lunardi C, Dolcino M, Peterlana D (2006). Antibodies against human cytomegalovirus in the pathogenesis of systemic sclerosis: a gene array approach. *PLoS Medicine*.

[10] Neidhart M, Kuchen S, Distler O (1999). Increased serum levels of antibodies against human cytomegalovirus and prevalence of autoantibodies in systemic sclerosis. *Arthritis and Rheumatism*.

[11] Jimenez SA, Artlett CM (2005). Microchimerism and systemic sclerosis. *Current Opinion in Rheumatology*.

[12] Tamby MC, Chanseaud Y, Guillevin L, Mouthon L (2003). New insights into the pathogenesis of systemic sclerosis. *Autoimmunity Reviews*.

[13] Corriveau MP, Boufaied I, Lessard J (2008). The fibrotic phenotype of systemic sclerosis fibroblasts varies with disease duration and severity of skin involvement: reconstitution of skin fibrosis development using a tissue engineering approach. *Journal of Pathology*.

[14] Abraham DJ, Varga J (2005). Scleroderma: from cell and molecular mechanisms to disease models. *Trends in Immunology*.

[15] Shi Y, Szalay MG, Paskar L, Boyer M, Singh B, Green DR (1990). Activation-induced cell death in T cell hybridomas is due to apoptosis. Morphologic aspects and DNA fragmentation. *Journal of Immunology*.

[16] Raff MC (1992). Social controls on cell survival and cell death. *Nature*.

[17] Hetts SW (1998). To Die or not to die: an overview of apoptosis and its role in disease. *Journal of the American Medical Association*.

[18] Savill J, Fadok V (2000). Corpse clearance defines the meaning of cell death. *Nature*.

[19] Kerr JF, Wyllie AH, Currie AR (1972). Apoptosis: a basic biological phenomenon with wide-ranging implications in tissue kinetics. *British Journal of Cancer*.

[20] Raff M (1998). Cell suicide for beginners. *Nature*.

[21] Cohen GM (1997). Caspases: the executioners of apoptosis. *Biochemical Journal*.

[22] Kroemer G, Reed JC (2000). Mitochondrial control of cell death. *Nature Medicine*.

[23] Adams JM, Cory S (1998). The Bcl-2 protein family: arbiters of cell survival. *Science*.

[24] Brenner C, Cadiou H, Vieira HLA (2000). Bcl-2 and Bax regulate the channel activity of the mitochondrial adenine nucleotide translocator. *Oncogene*.

[25] Taylor RC, Cullen SP, Martin SJ (2008). Apoptosis: controlled demolition at the cellular level. *Nature Reviews Molecular Cell Biology*.

[26] Subramanian RR, Masters SC, Zhang H, Fu H (2001). Functional conservation of 14-3-3 isoforms in inhibiting Bad-induced apoptosis. *Experimental Cell Research*.

[27] Liston P, Fong WG, Korneluk RG (2003). The inhibitors of apoptosis: there is more to life than Bcl2. *Oncogene*.

[28] Deveraux QL, Reed JC (1999). IAP family proteins - Suppressors of apoptosis. *Genes and Development*.

[29] Joza N, Susin SA, Daugas E (2001). Essential role of the mitochondrial apoptosis-inducing factor in programmed cell death. *Nature*.

[30] Ashkenazi A, Dixit VM (1998). Death receptors: signaling and modulation. *Science*.

[31] Thorburn A (2004). Death receptor-induced cell killing. *Cellular Signalling*.

[32] Wallach D, Varfolomeev EE, Malinin NL, Goltsev YV, Kovalenko AV, Boldin MP (1999). Tumor necrosis factor receptor and Fas signaling mechanisms. *Annual Review of Immunology*.

[33] Legler DF, Micheau O, Doucey MA, Tschopp J, Bron C (2003). Recruitment of TNF receptor 1 to lipid rafts is essential for TNF*α*-mediated NF-*κ*B activation. *Immunity*.

[34] Chan FKM, Chun HJ, Zheng L, Siegel RM, Bui KL, Lenardo MJ (2000). A domain in TNF receptors that mediates ligand-independent receptor assembty and signaling. *Science*.

[35] Siegel RM, Frederiksen JK, Zacharias DA (2000). Fas preassociation required for apoptosis signaling and dominant inhibition by pathogenic mutations. *Science*.

[36] Black RA, Rauch CT, Kozlosky CJ (1997). A metalloproteinase disintegrin that releases tumour-necrosis factor-*α* from cells. *Nature*.

[37] Wajant H, Pfizenmaier K, Scheurich P (2003). Tumor necrosis factor signaling. *Cell Death and Differentiation*.

[38] Wajant H, Pfizenmaier K, Scheurich P (2003). Non-apoptotic Fas signaling. *Cytokine and Growth Factor Reviews*.

[39] Aggarwal BB (2003). Signalling pathways of the TNF superfamily: a double-edged sword. *Nature Reviews Immunology*.

[40] Scaffidi C, Fulda S, Srinivasan A (1998). Two CD95 (APO-1/Fas) signaling pathways. *EMBO Journal*.

[41] Chinnalyan AM, O’Rourke K, Tewari M, Dixit VM (1995). FADD, a novel death domain-containing protein, interacts with the death domain of Fas and initiates apoptosis. *Cell*.

[42] Scaffidi C, Schmitz I, Krammer PH, Peter ME (1999). The role of c-FLIP in modulation of CD95-induced apoptosis. *Journal of Biological Chemistry*.

[43] Tschopp J, Irmler M, Thome M (1998). Inhibition of Fas death signals by FLIPs. *Current Opinion in Immunology*.

[44] Li H, Zhu H, Xu CJ, Yuan J (1998). Cleavage of BID by caspase 8 mediates the mitochondrial damage in the Fas pathway of apoptosis. *Cell*.

[45] Rubinfeld H, Seger R (2005). The ERK cascade: a prototype of MAPK signaling. *Molecular Biotechnology*.

[46] Dempsey PW, Doyle SE, He JQ, Cheng G (2003). The signaling adaptors and pathways activated by TNF superfamily. *Cytokine and Growth Factor Reviews*.

[47] Krueger A, Baumann S, Krammer PH, Kirchhoff S (2001). FLICE-inhibitory proteins: regulators of death receptor-mediated apoptosis. *Molecular and Cellular Biology*.

[48] Lieberman J (2003). The ABCs of granule-mediated cytotoxicity: new weapons in the arsenal. *Nature Reviews Immunology*.

[49] Yang X, Stennicke HR, Wang B (1998). Granzyme B mimics apical caspases: description of a unified pathway for trans-activation of executioner caspase-3 and -7. *Journal of Biological Chemistry*.

[50] Jiang HY, Wek SA, McGrath BC (2004). Activating transcription factor 3 is integral to the eukaryotic initiation factor 2 kinase stress response. *Molecular and Cellular Biology*.

[51] Kogel D, Schomburg R, Schürmann T (2003). The amyloid precursor protein protects PC12 cells against endoplasmic reticulum stress-induced apoptosis. *Journal of Neurochemistry*.

[52] Tabas I, Ron D (2011). Integrating the mechanisms of apoptosis induced by endoplasmic reticulum stress. *Nature Cell Biology*.

[53] Majno G, Joris I (1995). Apoptosis, oncosis, and necrosis: an overview of cell death. *American Journal of Pathology*.

[54] Galluzzi L, Kroemer G (2008). Necroptosis: a specialized pathway of programmed necrosis. *Cell*.

[55] Cheng J, Zhou T, Liu C (1994). Protection from Fas-mediated apoptosis by a soluble form of the Fas molecule. *Science*.

[56] Buhling F, Wille A, Röcken C (2005). Altered expression of membrane-bound and soluble CD95/Fas contributes to the resistance of fibrotic lung fibroblasts to FasL induced apoptosis. *Respiratory Research*.

[57] Tomokuni A, Otsuki T, Isozaki Y (1999). Serum levels of soluble Fas ligand in patients with silicosis. *Clinical and Experimental Immunology*.

[58] Otsuki T, Hayashi H, Nishimura Y (2011). Dysregulation of autoimmunity caused by silica exposure and alteration of Fas-mediated apoptosis in T lymphocytes derived from silicosis patients. *International Journal of Immunopathology and Pharmacology*.

[59] Tanaka T, Yoshimi M, Maeyama T, Hagimoto N, Kuwano K, Hara N (2002). Resistance to Fas-mediated apoptosis in human lung fibroblast. *European Respiratory Journal*.

[60] Thannickal VJ, Horowitz JC (2006). Evolving concepts of apoptosis in idiopathic pulmonary fibrosis. *Proceedings of the American Thoracic Society*.

[61] Budinger GRS, Mutlu GM, Eisenbart J (2006). Proapoptotic Bid is required for pulmonary fibrosis. *Proceedings of the National Academy of Sciences of the United States of America*.

[62] Kuwano K, Hagimoto N, Kawasaki M (1999). Essential roles of the Fas-Fas ligand pathway in the development of pulmonary fibrosis. *Journal of Clinical Investigation*.

[63] Kuwano K, Miyazaki H, Hagimoto N (1999). The involvement of Fas-Fas ligand pathway in fibrosing lung diseases. *American Journal of Respiratory Cell and Molecular Biology*.

[64] Ates A, Kinikli G, Turgay M, Duman M (2004). The levels of serum-soluble Fas in patients with rheumatoid arthritis and systemic sclerosis. *Clinical Rheumatology*.

[65] Bianchi T, Bardazzi F, Patrizi A (2000). Soluble fas levels in patients with systemic sclerosis. *Archives of Dermatological Research*.

[66] Bogatkevich GS, Gustilo E, Oates JC (2005). Distinct PKC isoforms mediate cell survival and DNA synthesis in thrombin-induced myofibroblasts. *American Journal of Physiology, Lung Cellular and Molecular Physiology*.

[67] Chabaud S, Corriveau M-P, Grodzicky T (2011). Decreased secretion of MMP by non-lesional late-stage scleroderma fibroblasts after selection via activation of the apoptotic fas-pathway. *Journal of Cellular Physiology*.

[68] Jelaska A, Korn JH (2000). Role of apoptosis and transforming growth factor *β*1 in fibroblast selection and activation in systemic sclerosis. *Arthritis and Rheumatism*.

[69] Santiago B, Galindo M, Rivero M, Pablos JL (2001). Decreased susceptibility to Fas-induced apoptosis of systemic sclerosis dermal fibroblasts. *Arthritis and Rheumatism*.

[70] Yamamoto T, Yokozeki H, Nishioka K (2007). Fas- and FasL-deficient mice are resistant to the induction of bleomycin-induced scleroderma. *Archives of Dermatological Research*.

[71] Yamamoto T, Nishioka K (2004). Possible role of apoptosis in the pathogenesis of bleomycin-induced scleroderma. *Journal of Investigative Dermatology*.

[72] Alaibac M, Berti E, Chizzolini C (2006). Role of cellular immunity in the pathogenesis of autoimmune skin diseases. *Clinical and Experimental Rheumatology*.

[73] Bendersky A, Markovits N, Bank I (2010). V*γ*9+ *γδ* T cells in systemic sclerosis patients are numerically and functionally preserved and induce fibroblast apoptosis. *Immunobiology*.

[74] Atamas SP, White B (2003). The role of chemokines in the pathogenesis of scleroderma. *Current Opinion in Rheumatology*.

[75] Giacomelli R, Cipriani P, Fulminis A (2001). Circulating *γ*/*δ* T lymphocytes from systemic sclerosis (SSc) patients display a T helper (Th) 1 polarization. *Clinical and Experimental Immunology*.

[76] De Palma R, Del Galdo F, Lupoli S, Altucci P, Abbate G, Valentini G (2006). Peripheral T lymphocytes from patients with early systemic sclerosis co-cultured with autologous fibroblasts undergo an oligoclonal expansion similar to that occurring in the skin. *Clinical and Experimental Immunology*.

[77] Parel Y, Aurrand-Lions M, Scheja A, Dayer JM, Roosnek E, Chizzolini C (2007). Presence of CD4+CD8+ double-positive T cells with very high interleukin-4 production potential in lesional skin of patients with systemic sclerosis. *Arthritis and Rheumatism*.

[78] Majone F, Olivieri S, Cozzi F (2009). Increased apoptosis in circulating lymphocyte cultures of anti-RNA polymerase III positive patients with systemic sclerosis. *Rheumatology International*.

[79] Cipriani P, Fulminis A, Pingiotti E (2006). Resistance to apoptosis in circulating *α*/*β* and *γ*/*δ* T lymphocytes from patients with systemic sclerosis. *Journal of Rheumatology*.

[80] Fang Y, Yu S, Ellis JS, Sharav T, Braley-Mullen H (2011). Comparison of sensitivity of Th1, Th2, and Th17 cells to Fas-mediated apoptosis. *Journal of Leukocyte Biology*.

[81] Fang Y, Braley-Mullen H (2008). Cultured murine thyroid epithelial cells expressing transgenic Fas-associated death domain-like interleukin-1*β* converting enzyme inhibitory protein are protected from Fas-mediated apoptosis. *Endocrinology*.

[82] Fang Y, Wei Y, DeMarco V, Chen K, Sharp GC, Braley-Mullen H (2007). Murine FLIP transgene expressed on thyroid epithelial cells promotes resolution of granulomatous experimental autoimmune thyroiditis in DBA/1 mice. *American Journal of Pathology*.

[83] Sgonc R, Gruschwitz MS, Boeck G, Sepp N, Gruber J, Wick G (2000). Endothelial cell apoptosis in systemic sclerosis is induced by antibody-dependent cell-mediated cytotoxicity via CD95. *Arthritis and Rheumatism*.

[84] Ahmed SS, Tan FK, Arnett FC, Jin L, Geng YJ (2006). Induction of apoptosis and fibrillin 1 expression in human dermal endothelial cells by scleroderma sera containing anti-endothelial cell antibodies. *Arthritis and Rheumatism*.

[85] Lunardi C, Bason C, Navone R (2000). Systemic sclerosis immunoglobulin G autoantibodies bind the human cytomegalovirus late protein UL94 and induce apoptosis in human endothelial cells. *Nature Medicine*.

[86] Barnes TC, Spiller DG, Anderson ME, Edwards SW, Moots RJ (2010). Endothelial activation and apoptosis mediated by neutrophil-dependent interleukin 6 trans-signalling: a novel target for systemic sclerosis?. *Annals of the Rheumatic Diseases*.

[87] del Papa N, Quirici N, Scavullo C (2010). Antiendothelial cell antibodies induce apoptosis of bone marrow endothelial progenitors in systemic sclerosis. *Journal of Rheumatology*.

[88] Zhu S, Evans S, Yan B (2008). Transcriptional regulation of Bim by FOXO3a and Akt mediates scleroderma serum-induced apoptosis in endothelial progenitor cells. *Circulation*.

[89] Distler JHW, Akhmetshina A, Dees C (2011). Induction of apoptosis in circulating angiogenic cells by microparticles. *Arthritis and Rheumatism*.

[90] Distler JHW, Huber LC, Gay S, Distler O, Pisetsky DS (2006). Microparticles as mediators of cellular cross-talk in inflammatory disease. *Autoimmunity*.

[91] Bielecki M, Kowal K, Lapinska A (2009). Diminished production of TWEAK by the peripheral blood mononuclear cells is associated with vascular involvement in patients with systemic sclerosis. *Folia Histochemica et Cytobiologica*.

[92] Yanaba K, Yoshizaki A, Muroi E (2009). Elevated circulating TWEAK levels in systemic sclerosis: association with lower frequency of pulmonary fibrosis. *Journal of Rheumatology*.

[93] Brinckmann J, Kim S, Wu J (2005). Interleukin 4 and prolonged hypoxia induce a higher gene expression of lysyl hydroxylase 2 and an altered cross-link pattern: important pathogenetic steps in early and late stage of systemic scleroderma?. *Matrix Biology*.

[94] Beyer C, Schett G, Gay S, Distler O, Distler JH (2009). Hypoxia. Hypoxia in the pathogenesis of systemic sclerosis. *Arthritis Research &amp; Therapy*.

[95] Hagimoto N, Kuwano K, Nomoto Y, Kunitake R, Hara N (1997). Apoptosis and expression of Fas/Fas ligand mRNA in bleomycin-induced pulmonary fibrosis in mice. *American Journal of Respiratory Cell and Molecular Biology*.

[96] Chapman HA (1999). A Fas pathway to pulmonary fibrosis. *Journal of Clinical Investigation*.

[97] Hoyles RK, Khan K, Shiwen X (2008). Fibroblast-specific perturbation of transforming growth factor *β* signaling provides insight into potential pathogenic mechanisms of scleroderma-associated lung fibrosis: exaggerated response to alveolar epithelial injury in a novel mouse model. *Arthritis and Rheumatism*.

[98] Kuwano K, Kunitake R, Kawasaki M (1996). p21(Waf1/Cip1/Sdi1) and p53 expression in association with DNA strand breaks in idiopathic pulmonary fibrosis. *American Journal of Respiratory and Critical Care Medicine*.

[99] Golan-Gerstl R, Wallach-Dayan SB, Amir G, Breuer R (2007). Epithelial cell apoptosis by Fas ligand-positive myofibroblasts in lung fibrosis. *American Journal of Respiratory Cell and Molecular Biology*.

[100] Aden N, Shiwen X, Aden D (2008). Proteomic analysis of scleroderma lesional skin reveals activated wound healing phenotype of epidermal cell layer. *Rheumatology*.

[101] Aden N, Nuttall A, Shiwen X (2010). Epithelial cells promote fibroblast activation via IL-1*α* in systemic sclerosis. *Journal of Investigative Dermatology*.

[102] Bellemare J, Roberge CJ, Bergeron D, Lopez-Vallé CA, Roy M, Moulin VJ (2005). Epidermis promotes dermal fibrosis: role in the pathogenesis of hypertrophic scars. *Journal of Pathology*.

[103] Larochelle S, Langlois C, Thibault I, Lopez-Vallé CA, Roy M, Moulin V (2004). Sensitivity of myofibroblasts to H2O2-mediated apoptosis and their antioxidant cell network. *Journal of Cellular Physiology*.

[104] Moulin V, Larochelle S, Langlois C, Thibault I, Lopez-Vallé CA, Roy M (2004). Normal skin wound and hypertrophic scar myofibroblasts have differential responses to apoptotic inductors. *Journal of Cellular Physiology*.

[105] Kim JY, Kwok SK, Hur KH (2008). Up-regulated macrophage migration inhibitory factor protects apoptosis of dermal fibroblasts in patients with systemic sclerosis. *Clinical and Experimental Immunology*.

[106] Jun JB, Kuechle M, Min J (2005). Scleroderma fibroblasts demonstrate enhanced activation of Akt (protein kinase B) in situ. *Journal of Investigative Dermatology*.

[107] Horowitz JC, Rogers DS, Sharma V (2007). Combinatorial activation of FAK and AKT by transforming growth factor-*β*1 confers an anoikis-resistant phenotype to myofibroblasts. *Cellular Signalling*.

[108] Mimura Y, Ihn H, Jinnin M, Asano Y, Yamane K, Tamaki K (2005). Constitutive phosphorylation of focal adhesion kinase is involved in the myofibroblast differentiation of scleroderma fibroblasts. *Journal of Investigative Dermatology*.

[109] Simonini G, Cerinic MM, Generini S (1999). Oxidative stress in systemic sclerosis. *Molecular and Cellular Biochemistry*.

[110] Avouac J, Borderie D, Ekindjian OG, Kahan A, Allanore Y (2010). High DNA oxidative damage in systemic sclerosis. *Journal of Rheumatology*.

[111] Jelaska A, Arakawa M, Broketa G, Korn JH (1996). Heterogeneity of collagen synthesis in normal and systemic sclerosis skin fibroblasts: increased proportion of high collagen-producing cells in systemic sclerosis fibroblasts. *Arthritis and Rheumatism*.

[112] Botstein GR, Sherer GK, Leroy EC (1982). Fibroblast selection in scleroderma. An alternative model of fibrosis. *Arthritis and Rheumatism*.

[113] Needleman BW, Ordonez JV, Taramelli D, Alms W, Gayer K, Choi J (1990). In vitro identification of a subpopulation of fibroblasts that produces high levels of collagen in scleroderma patients. *Arthritis and Rheumatism*.

[114] Jun JB, Kuechle M, Harlan JM, Elkon KB (2003). Fibroblast and endothelial apoptosis in systemic sclerosis. *Current Opinion in Rheumatology*.

[115] Shanahan WR, Korn JH (1982). Cytotoxic activity of sera from scleroderma and other connective tissue diseases. Lack of cellular and disease specificity. *Arthritis and Rheumatism*.

[116] Liu K, McDuffie E, Abrams SI (2003). Exposure of human primary colon carcinoma cells to anti-Fas interactions influences the emergence of pre-existing Fas-resistant metastatic subpopulations. *Journal of Immunology*.

[117] de Palma R, D’aiuto E, Vettori S, Cuoppolo P, Abbate G, Valentini G (2010). Peripheral T cells from patients with early systemic sclerosis kill autologous fibroblasts in co-culture: is T-cell response aimed to play a protective role?. *Rheumatology*.

[118] Laplante P, Sirois I, Raymond MA (2010). Caspase-3-mediated secretion of connective tissue growth factor by apoptotic endothelial cells promotes fibrosis. *Cell Death and Differentiation*.

[119] Holmes A, Abraham DJ, Sa S, Shiwen X, Black CM, Leask A (2001). CTGF and SMADs, maintenance of scleroderma phenotype is independent of SMAD signaling. *Journal of Biological Chemistry*.

[120] Laplante P, Raymond MA, Gagnon G (2005). Novel fibrogenic pathways are activated in response to endothelial apoptosis: implications in the pathophysiology of systemic sclerosis. *Journal of Immunology*.

[121] Nacu N, Luzina IG, Highsmith K (2008). macrophages Produce TGF-*β*-induced (*β*-h3) following ingestion of apoptotic cells and regulate MMP14 levels and collagen turnover in fibroblasts. *Journal of Immunology*.

[122] Mimura Y, Ihn H, Jinnin M, Asano Y, Yamane K, Tamaki K (2005). Constitutive thrombospondin-1 overexpression contributes to autocrine transforming growth factor-*β* signaling in cultured scleroderma fibroblasts. *American Journal of Pathology*.

[123] Moodley Y, Rigby P, Bundell C (2003). Macrophage recognition and phagocytosis of apoptotic fibroblasts is critically dependent on fibroblast-derived thrombospondin 1 and CD36. *American Journal of Pathology*.

[124] Mott JL, Kobayashi S, Bronk SF, Gores GJ (2007). mir-29 regulates Mcl-1 protein expression and apoptosis. *Oncogene*.

[125] Maurer B, Stanczyk J, Jüngel A (2010). MicroRNA-29, a key regulator of collagen expression in systemic sclerosis. *Arthritis and Rheumatism*.

[126] Mott JL, Kurita S, Cazanave SC, Bronk SF, Werneburg NW, Fernandez-Zapico ME (2010). Transcriptional suppression of mir-29b-1/mir-29a promoter by c-Myc, hedgehog, and NF-kappaB. *Journal of Cellular Biochemistry*.

[127] Koch AE, Kronfeld-Harrington LB, Szekanecz Z (1993). In situ expression of cytokines and cellular adhesion molecules in the skin of patients with systemic sclerosis. Their role in early and late disease. *Pathobiology*.

[128] Becvar R, Hulejová H, Braun M, Štork J (2007). Collagen degradation products and proinflammatory cytokines in systemic and localized scleroderma. *Folia Biologica*.

[129] Horikawa M, Hasegawa M, Komura K (2005). Abnormal natural killer cell function in systemic sclerosis: altered cytokine production and defective killing activity. *Journal of Investigative Dermatology*.

[130] Kawaguchi Y, Hara M, Wright TM (1999). Endogenous IL-1*α* from systemic sclerosis fibroblasts induces IL-6 and PDGF-A. *Journal of Clinical Investigation*.

[131] Sato S, Hasegawa M, Takehara K (2001). Serum levels of interleukin-6 and interleukin-10 correlate with total skin thickness score in patients with systemic sclerosis. *Journal of Dermatological Science*.

[132] Xiao LZ, Topley N, Ito T, Phillips A (2005). Interleukin-6 regulation of transforming growth factor (TGF)-*β* receptor compartmentalization and turnover enhances TGF-*β*1 signaling. *Journal of Biological Chemistry*.

[133] Moodley YP, Misso NLA, Scaffidi AK (2003). Inverse effects of interleukin-6 on apoptosis of fibroblasts from pulmonary fibrosis and normal lungs. *American Journal of Respiratory Cell and Molecular Biology*.

[134] Legembre P, Daburon S, Moreau P (2005). Amplification of Fas-mediated apoptosis in type II cells via microdomain recruitment. *Molecular and Cellular Biology*.

[135] Legembre P, Daburon S, Moreau P, Moreau JF, Taupin JL (2006). Modulation of Fas-mediated apoptosis by lipid rafts in T lymphocytes. *Journal of Immunology*.

[136] Ramaswamy M, Dumont C, Cruz AC (2007). Cutting edge: Rac GTPases sensitize activated T cells to die via Fas. *Journal of Immunology*.

[137] Beneteau M, Pizon M, Chaigne-Delalande B (2008). Localization of Fas/CD95 into the lipid rafts on down-modulation of the phosphatidylinositol 3-kinase signaling pathway. *Molecular Cancer Research*.

[138] Muppidi JR, Siegel RM (2004). Ligand-independent redistribution of Fas (CD95) into lipid rafts mediates clonotypic T cell death. *Nature Immunology*.

[139] Aouad SM, Cohen LY, Sharif-Askari E, Haddad EK, Alam A, Sekaly RP (2004). Caspase-3 is a component of Fas death-inducing signaling complex in lipid rafts and its activity is required for complete caspase-8 activation during Fas-mediated cell death. *Journal of Immunology*.

[140] Wei J, Bhattacharyya S, Tourtellotte WG, Varga J (2011). Fibrosis in systemic sclerosis: emerging concepts and implications for targeted therapy. *Autoimmunity Reviews*.

[141] Shukla S, Fujita KI, Xiao Q, Liao Z, Garfield S, Srinivasula SM (2010). A shear stress responsive gene product PP1201 protects against Fas-mediated apoptosis by reducing Fas expression on the cell surface. *Apoptosis*.

[142] Varadhachary AS, Edidin M, Hanlon AM, Peter ME, Krammer PH, Salgame P (2001). Phosphatidylinositol 3′-kinase blocks CD95 aggregation and caspase-8 cleavage at the death-inducing signaling complex by modulating lateral diffusion of CD95. *Journal of Immunology*.

[143] Derk CT (2007). Associations of breast cancer development in patients with systemic sclerosis: an exploratory study. *Clinical Rheumatology*.

[144] Behjati S, Frank MH (2009). The effects of tamoxifen on immunity. *Current Medicinal Chemistry*.

[145] Shin SM, Kim SG (2009). Inhibition of arachidonic acid and iron-induced mitochondrial dysfunction and apoptosis by oltipraz and novel 1,2-dithiole-3-thione congeners. *Molecular Pharmacology*.

[146] Ito T, Williams JD, Fraser DJ, Phillips AO (2004). Hyaluronan regulates transforming growth factor-*β*1 receptor compartmentalization. *Journal of Biological Chemistry*.

[147] Tourkina E, Richard M, Gööz P (2008). Antifibrotic properties of caveolin-1 scaffolding domain in vitro and in vivo. *American Journal of Physiology, Lung Cellular and Molecular Physiology*.

[148] Qian N, Ueno T (2010). Is dysfunction of caveolin-1 a link between systemic sclerosis and breast cancer, opening a window on both etiologies?. *Archives of Medical Research*.

[149] Tourkina E, Gooz P, Oates JC, Ludwicka-Bradley A, Silver RM, Hoffman S (2004). Curcumin-induced apoptosis in scleroderma lung fibroblasts: role of protein kinase C*ε*. *American Journal of Respiratory Cell and Molecular Biology*.

[150] Miller M, Chen S, Woodliff J, Kansra S (2008). Curcumin (diferuloylmethane) inhibits cell proliferation, induces apoptosis, and decreases hormone levels and secretion in pituitary tumor cells. *Endocrinology*.

[151] Wang Z, Desmoulin S, Banerjee S (2008). Synergistic effects of multiple natural products in pancreatic cancer cells. *Life Sciences*.

[152] Majumdar APN, Banerjee S, Nautiyal J (2009). Curcumin synergizes with resveratrol to inhibit colon cancer. *Nutrition and Cancer*.

[153] Singh S, Khar A (2006). Biological effects of curcumin and its role in cancer chemoprevention and therapy. *Anti-Cancer Agents in Medicinal Chemistry*.

[154] Singh N, Shrivastav A, Sharma RK (2009). Curcumin induces caspase and calpain-dependent apoptosis in HT29 human colon cancer cells. *Molecular Medicine Reports*.

[155] Fulda S (2011). Modulation of apoptosis by natural products for cancer therapy. *Planta Medica*.

[156] Fulda S, Debatin KM (2006). Resveratrol modulation of signal transduction in apoptosis and cell survival: a mini-review. *Cancer Detection and Prevention*.

[157] Simonini G, Alberto P, Sergio G, Fernanda F, Marco MC (2000). Emerging potentials for an antioxidant therapy as a new approach to the treatment of systemic sclerosis. *Toxicology*.

[158] Lee ES, Kim YA, Kwon HJ, Bang D, Lee S, Sohn S (2004). Thalidomide upregulates macrophage inflammatory protein-1*α* in a herpes simplex virus-induced Behçet’s disease-like animal model. *Archives of Dermatological Research*.

[159] Yasui K, Kobayashi N, Yamazaki T, Agematsu K (2005). Thalidomide as an immunotherapeutic agent: the effects on neutrophil-mediated inflammation. *Current Pharmaceutical Design*.

[160] Gockel HR, Lügering A, Heidemann J (2004). Thalidomide Induces Apoptosis in Human Monocytes by Using a Cytochrome c-Dependent Pathway. *Journal of Immunology*.

[161] Huber SA, Kupperman J, Newell MK (1999). Estradiol prevents and testosterone promotes Fas-dependent apoptosis in CD4+ Th2 cells by altering Bcl 2 expression. *Lupus*.

[162] Ghobrial IM, Witzig TE, Adjei AA (2005). Targeting apoptosis pathways in cancer therapy. *Ca-A Cancer Journal for Clinicians*.

